# Projected income data under different shared socioeconomic pathways for Washington state

**DOI:** 10.1038/s41597-023-02906-5

**Published:** 2024-01-18

**Authors:** Heng Wan, Sumitrra Ganguli, Narmadha Meenu Mohankumar, Milan Jain, Kyle Wilson, David Anderson

**Affiliations:** 1 Earth Systems Predictability & Resiliency Group, Pacific Northwest National Laboratory, Richland, WA 99352 USA; 2 Economics, Policy & Institutional Support Group, Pacific Northwest National Laboratory, Richland, WA 99352 USA; 3 Math, Stats & Data Science Group, Pacific Northwest National Laboratory, Richland, WA 99352 USA; 4 Optimization & Control Group, Pacific Northwest National Laboratory, Richland, WA 99352 USA; 5 Risk & Environmental Assessment Group, Pacific Northwest National Laboratory, Richland, WA 99352 USA

**Keywords:** Databases, Society

## Abstract

High-resolution income projections under different Shared Socioeconomic Pathways (SSPs) are essential for the climate change research communities to devise climate change adaptation and mitigation strategies. To generate income projections for Washington state, we obtain state-level GDP per capita projections and convert them into projected annual household income. The resulting state-level income projections are subsequently downscaled to the census block-level based on the Longitudinal Origin-Destination Employment Statistics (LODES) dataset. For accuracy assessment, we downscale historical income data from state- level to block- and block group-level and compare the downscaled results against the actual income data from LODES. County-level accuracy assessment is also conducted based on American Community Survey. The results demonstrate a good agreement (Average R^2^ of 0.67, 0.8, and 0.99 for block-, block group-, and county-level, respectively) between the downscaled income data and the reference data, thereby validating the methodology employed. Our approach is applicable to other states for income projections, which can be utilized by a broader audience, including those involved in demographic analysis, economic research, and urban planning.

## Background & Summary

Income is an important data input and is increasingly used in many research fields, such as demographic analysis, economic research, urban planning, market research, and housing studies^1–5^. In the United States, income data are regularly collected by American Community Survey (ACS), the largest survey in the United States that collects detailed social, economic, housing, and demographic information by sampling about 3 million addresses every year^6^. ACS provides income data across different census geographic levels (e.g., block group, census tract, county, state) and different temporal resolutions (e.g., annual data, 5-year average data) in various forms, including aggregate income, median household income, number of households by 16 income categories, etc^7^. However, the ACS estimated income data for small areas (e.g., block groups, census tracts) is subject to significant uncertainty caused by sampling error, which often renders the data too imprecise to be utilized^8^. On the other side, the US Census Longitudinal Employer-Household Dynamics (LEHD) Origin-Destination Employment Statistics (LODES) dataset^9^ provides block-level job-related information, including income data (employee compensation), directly compiled from administrative records, which eliminates the issues of sampling errors^10^. LODES provides three different data types, including Work Area Characteristics (WAC), Residence Area Characteristics (RAC), and Origin-Destination (OD). WAC and RAC respectively provide job information by work census block and residence census block, while OD provides job information associated with both a residence census block and a work census block. The income data in the LODES dataset are shown as the number of jobs by three different wage categories ( ≤ $1,250/month, $1,251 ~ $3,333/month, and > $3,333/month)^11^. One major difference between the ACS income data and the LODES income data is that LODES focuses on employed individuals, while ACS covers the entire population, including employed individuals, self-employed persons, retirees, and others^7,12^. Therefore, some income categories, such as self-employment income, pensions, interests and dividends, and rental income, are included in ACS but not in LODES.

The availability of historical and current income data at high spatial and temporal resolution has provided valuable information for research, however, one notable gap in current research and analysis is the limited estimation of future income projections at a similar high resolution. As climate change is projected to accelerate in the future, extreme weather events (e.g., heat wave, flood, drought, etc.) will become more frequent and severe, making humans more vulnerable to them^13^. Quantifying future demographics in high resolution under different Shared Socioeconomic Pathways (SSPs) scenarios can help to assess climate change vulnerabilities, making it useful for designing climate change adaptation and mitigation strategies on a more detailed and granular scale^14^. Numerous research has been done to facilitate this, for example, Jones and O’Neill produced global one-eighth degree population grids for every decade from 2000 to 2100 under different SSPs^15^. As an important indicator for social vulnerability, income projections play a crucial role in depicting future income trajectories and identifying possible disadvantaged communities that may be vulnerable in future climate change scenarios, which is vital for devising climate change adaptation and mitigation strategies^16,17^. Nevertheless, it’s important to recognize that future incomes in certain spaces may be endogenous to future economic conditions, including economic growth and inequality, as individuals and households can relocate in response to changing circumstances.

Our study generates 3-binned block- and block group- level income projections for every five years between 2020 and 2100 under different SSPs for Washington state. These income projections are estimated as number of households by three different income bins consistent with the LODES income categories. Our study provides granular income data for climate research projections, which are vital for devising climate change adaptation and mitigation strategies. For instance, these income projections for Washington state are utilized in the Grid Operations, Decarbonization, Environmental and Energy Equity Platform (GODEEEP), which intends to model the US energy system interactions across scales under decarbonization and assess its impact on environmental and energy equity. Specifically, block-level income projection is employed to predict disadvantaged communities (also referred as DAC)^18^. The US Council on Environmental Quality’s Justice40 initiatives have recently defined DAC at the census tract-level, enabling an understanding of which areas are currently experiencing the benefits or lack thereof from climate and energy investments. By incorporating block- and block group-level income projections into our spatial disaggregation analysis, we can estimate DAC at finer spatial resolution.

**Fig. 1:**
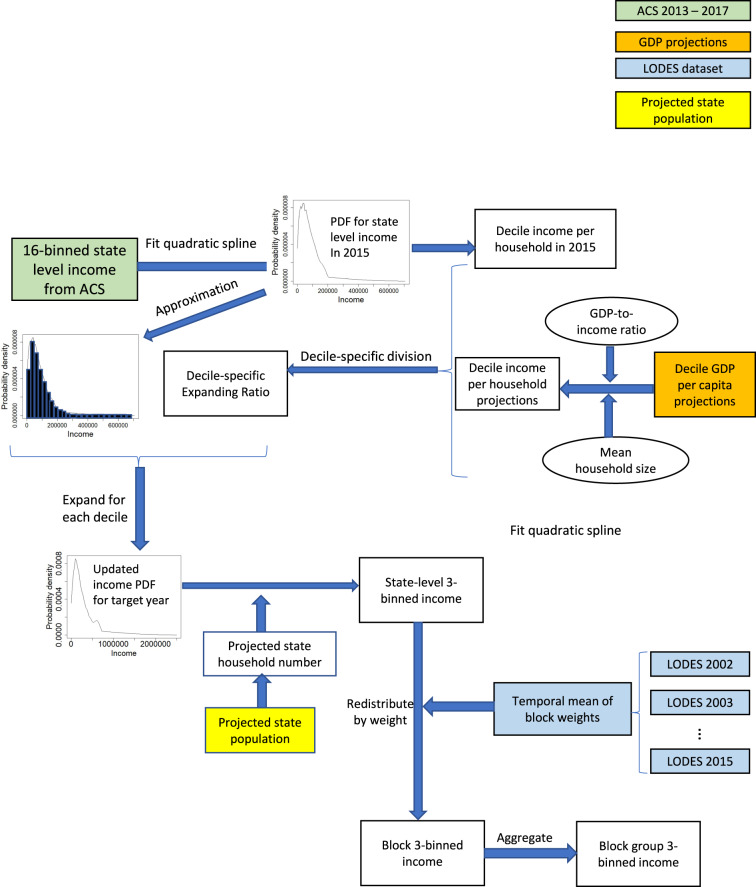
Workflow for income reconciliation.

Figure 1 shows the workflow of income reconciliation. More detailed data generation steps are described in the method section. Our approach could also be applied to other states to generate income projections using similar data.

## Methods

We obtain the ACS 2013–2017 state-level population, number of households, aggregate income, and 16-binned income data from the Integrated Public Use Microdata Series (IPUMS)^19^. ACS 2013–2017 contains 5-year estimates from data collected during 2013–2017 period, which represents the socioeconomic conditions for our baseline year of 2015. To derive the state-level mean household size and average household income, we respectively divide the aggregated state-level income and population by the state-level number of households. The resulting state-level mean household size is used for the calculation of the projected decile income per household while the derived state-level mean household income is used to estimate the state-level income probability density function (PDF) in the baseline year of 2015. Hippel *et al*. introduced a method of fitting nonparametric continuous distribution based on binned income data by interpolating the corresponding cumulative distribution function^20^. The resulted PDF can reproduce the bin counts accurately, and the estimation accuracy improves when the interpolation method is constrained to match a known mean. We utilize the “binsmooth” package^20^ in R and implement the interpolation method using splines to estimate the baseline state-level income PDF based on the state-level 16-binned income data, with the calculated state-level mean household income serving as the mean constraint. Subsequently, we estimate the future projected income PDFs using the derived baseline state-level income PDF to reflect corresponding changes. Furthermore, based on the estimated baseline state-level income PDF, we calculate the baseline state-level decile household income in 2015.

We obtain projected decile real GDP per capita for future years (G_i_) under different SSPs^21^ (Only SSP2, SSP3, and SSP5 are available in this product). We convert projected decile GDP per capita to projected decile income per household (M_i_) using the formula:

$${{\rm{M}}}_{{\rm{i}}}={{\rm{G}}}_{{\rm{i}}}\ast {\rm{H}}/{\rm{R}},$$

Where M_i_ is the projected i-ith decile income per household, G_i_ is the projected i-th decile GDP per capita, H is the mean household size, and R is the GDP-to-income ratio. This conversion formula assumes that the GDP-to-income ratio and the mean household size remain constant over time, which aligns with the historic observations (Please refer to Supplementary Information for the validation details regarding the constant mean household size and GDP-to-income ratio assumptions).

The derived projected decile income per household is compared with the baseline decile income per household in 2015 (B_i_) to calculate decile-specific expanding ratios (ER_i_) based on the formula:

$${{\rm{ER}}}_{{\rm{i}}}={{\rm{M}}}_{{\rm{i}}}/{{\rm{B}}}_{{\rm{i}}},$$

These decile-specific expanding ratios reflect income changes for each decile, and they are further used to update the income PDF in the baseline year of 2015.

To update the income PDF from the baseline year of 2015 to the projected years, we first discretize the continuous income PDF of 2015 into bins with an equal width of $100. We calculate an updated point for each bin by multiplying the midpoint of its x-axis by the corresponding decile-specific expanding ratio. For instance, the $10,000 - $10,100 bin belongs to the first income decile, with a corresponding expanding ratio of 1.2. Its midpoint at $10,050 is used as its representation and is then multiplied by the expanding ratio, resulting in an updated point with an x-axis value of $12,060, while the y-axis value (probability) remains unchanged. Based on all the updated points, we fit a spline function. We subsequently normalize this fitted spline function to ensure that the cumulative y values sum up to 1 (Sum of probability equals to 1). This normalized spline function serves as the income PDF projection.

We then obtain state-level population projections from Zoraghein and O’Neil *et al.*^22^ and calculate state-level number of households projections based on the following formula:

$${\rm{N}}={\rm{P}}/{\rm{H}},$$

where N represents the state-level number of households projection, P is the projected state-level population projection, and H is the mean household size. We integrate the income PDF projection with the corresponding state-level number of households projection to derive the projected state-level income data, which is represented as the number of households in three income bins defined by LODES.

However, before integration, we need to first address the mismatch between the income units used in the income PDF projections and the LODES data. The income PDF projections represent income as annual household income, while LODES defines three income bins as monthly wages per person. Therefore, we convert the income unit in LODES to annual household income based on the following formula:

$${\rm{Income}}={\rm{Wage}}\ast {\rm{E}}\ast 12\;{\rm{month}},$$

where Income represents annual household income, Wage is monthly wage per person, and E is the average number of employees per household, calculated as a temporal mean based on LODES data from 2002 to 2015. As a result, the three income bins defined by LODES (≤$1,250/month/person, $1,251~$3,333/month/person, and >$3,333/month/person) are transformed to annual household income bins (≤$18,150/year/household, $18,150~$48,396/year/household, and >$48,396/year/household).

Once we obtain income projections at the state-level, we further downscale them to block-level using the following formula:

$${{\rm{BK}}}_{{\rm{i}},{\rm{j}}}={{\rm{S}}}_{{\rm{i}}}\ast {{\rm{W}}}_{{\rm{i}},{\rm{j}}.}$$

Here, BK_i,j_ is the downscaled block-level number of households for the i-th income bin and the j-th census block, S_i_ is the state-level number of households in the i-th income bin, and W_i, j_ is the redistribution weight for the j-th census block in terms of the i-th income bin. W_i, j_ is a temporal mean weight and can be calculated by the following formula:

$${{\rm{W}}}_{{\rm{i}},{\rm{j}}}=\frac{1}{14}\mathop{\sum }\limits_{{\rm{k}}=2002}^{2015}{{\rm{BK}}}_{{\rm{i}},{\rm{j}},{\rm{k}},}/{{\rm{S}}}_{{\rm{i}},{\rm{k}},}$$

where BK_i, j,k_ is the number of households for the i-th income bin for the j-th census block in historical year k, and S_i, k_ is the state total number of households for i-th income bin in historical year k. This downscaling procedure assumes that the block-level weight remains relatively stable over years. Once the block-level income projections are calculated, we aggregate them into block group-level.

## Data Records

Income projections for Washington state (under census geographic boundary 2020) for every five years from 2020 to 2100 under different SSPs (SSP2, SSP3, and SSP5) are publicly available at Zenodo repository^23^ (Version 4). The files “bg_binned_income_proj_rounded.csv” and “bk_binned_income_proj_rounded.csv” contain income projections at the block group- and block-level, respectively. Income projection data are shown as the projected number of households for each of the three different income categories. In the data file, “GISJOIN” is the unique identifier for each block (or block group). Each number of households projection is stored in a column, where the first four characters of the column name represent the projection year (e.g., 2020, 2030), and the remaining characters indicate the income category (Income1 represents annual household income less than $18,150, Income2 represents annual household income between $18,150 and $48,396, and Income3 represents annual household income greater than $48,396). For example, “2020SSP2Income1” indicates the number of households projection for the first income bin in 2020 under SSP2.

## Technical Validation

In this study, we assume that the GDP-to-income ratio, the mean household size, and the average number of employees per household are constant over time for generating state-level income projections. To further downscale these projections to the block level, we assume that the proportion of households within each block, categorized by income, remains stable to the state’s total households in the same income category over time. To validate our method, we apply it to downscale historical income data from state-level to block- and block group-level, and then compare the downscaled results against the actual income data.

Specifically, three-binned income data at the state-level for years between 2016 and 2020 are obtained by aggregating block-level LODES income data^9^. Then, state-level three-binned incomes are downscaled to block-level based on the income-bin-specific block-level population weights based on LODES 2002–2015. To obtain block group-level income data, the downscaled block-level income data are aggregated to block group-level. The downscaled block/block group-level income data are compared with the actual block/block group-level LODES income data. We calculate the R^2^, median absolute percentage error (MdAPE), Households Placed Incorrectly (HPI), and Percent of Households Placed Incorrectly (PHPI) for each income bin and each validation year between 2016 and 2020. HPI is calculated based on the following equation:

$${\rm{HPl}}=\frac{{{\sum }}_{{i}}^{{n}}| {{r}}_{{i}}-{{a}}_{{i}}| }{2},$$

where r_i_ and a_i_ are the downscaled number of households and actual number of households for certain income bin at the i^th^ block/block group, respectively. The division by two corrects for the double counting of the incorrectly placed households in the block/block group where they actually reside and the block/block group where they were mistakenly placed^24^. PHPI is calculated as the percentage of HPI over the state total number of households.

Tables 1 and 2 show the accuracy assessment results for block-level and block group-level income data, respectively. The downscaled block group-level income data outperforms the downscaled block-level income data across all validation years. In addition, these two tables demonstrate the stability of the accuracy for each income bin across different validation years, except for year 2020 where both block- and block group-level income data showed deteriorated accuracy. The observed deteriorated accuracy for 2020 can be attributed to various factors. One significant contributor is the population migrations induced by COVID-19 in 2020, which led to considerable variations in population distributions in 2020 compared with past years^25^. Additionally, the way the LODES data were compiled could also contribute to this deteriorated accuracy in 2020. Specifically, LODES data for 2016–2019 used the census block boundary from 2010, whereas LODES 2020 was compiled based on the census block boundary from 2020. To align the original LODES data from 2016–2019 with the 2020 census geographic boundary, the US Census Bureau employed areal interpolation^11^. However, this areal interpolation step could introduce additional uncertainty for those years, resulting a larger divergence from LODES 2020, which directly utilized the census block boundary of 2020.

**Table 1:** Accuracy assessment for downscaled block-level income data.

	R2	MdAPE	PPI	PPPI
2016				
Income bin 1	0.8	33%	121,546	19.92%
Income bin 2	0.84	33%	170,968	17.47%
Income bin 3	0.84	33%	260,281	16.29%
2017				
Income bin 1	0.79	36%	119,850	20.38%
Income bin 2	0.83	33%	173,062	17.68%
Income bin 3	0.83	33%	276,504	16.39%
2018				
Income bin 1	0.79	36%	117,504	20.66%
Income bin 2	0.83	33%	173,450	18.11%
Income bin 3	0.83	33%	303,989	16.68%
2019				
Income bin 1	0.79	38%	116,153	20.93%
Income bin 2	0.82	33%	170,246	18.53%
Income bin 3	0.83	33%	314,115	16.94%
2020				
Income bin 1	0.68	50%	151732	24.25%
Income bin 2	0.7	48%	187,263	23.35%
Income bin 3	0.71	40%	382936	21.00%

**Table 2:** Accuracy assessment for downscaled block group-level income data.

	R2	MdAPE	PPI	PPPI
2016				
Income bin 1	0.68	13%	49,540	8.12%
Income bin 2	0.76	13%	74,536	7.61%
Income bin 3	0.78	13%	132,674	8.30%
2017				
Income bin 1	0.66	14%	49,754	8.46%
Income bin 2	0.74	13%	78,391	8.01%
Income bin 3	0.77	14%	145,558	8.63%
2018				
Income bin 1	0.64	14%	49,362	8.68%
Income bin 2	0.71	14%	82,940	8.66%
Income bin 3	0.74	15%	169,506	9.30%
2019				
Income bin 1	0.62	14%	49,816	8.97%
Income bin 2	0.69	15%	82,416	8.97%
Income bin 3	0.72	16%	181,726	9.80%
2020				
Income bin 1	0.41	18%	72676	11.55%
Income bin 2	0.5	19%	96,348	11.94%
Income bin 3	0.54	18%	220750	12.00%

Besides accuracy assessment for historical years between 2016 and 2019 based on LODES dataset, we extend the evaluation to our income projection product in 2020 under SSP2 (Business-as-usual scenario) based on the ACS dataset. Unlike the LODES-based evaluations, the ACS-based accuracy assessment is conducted at a coarser resolution of county-level due to the high sampling errors associated with the ACS income data at finer spatial resolutions^8^. As ACS 1-year estimate of income for 2020 is not available due to covid^26^, we download ACS 2019 and ACS 2021 and then calculate their mean to represent ACS income data in 2020. Then, we aggregate our income projection in 2020 under SSP2 to the county-level, followed by the comparison with county-level ACS income data in 2020. Table 3 shows the accuracy assessment result. The result reveals that the county-level accuracy assessment exhibits superior performance, characterized by higher R^2^ values and lower error values.

**Table 3:** County-level accuracy assessment for 2020.

	R2	MdPE	PPI	PPPI
Income bin 1	0.99	15%	13,412	5.48%
Income bin 2	0.98	11%	44,975	8.47%
Income bin 3	0.99	15%	134,782	6.72%

### Supplementary information

Supplementary Information

## Data Availability

Code for generating block/block group-level 3-binned income for the projected years under different SSPs can be found at GitHub (https://github.com/crystalandwan/Income-Reconciliation.git).
