# Publisher Correction: Projected income data under different shared socioeconomic pathways for Washington state

**DOI:** 10.1038/s41597-024-02992-z

**Published:** 2024-01-29

**Authors:** Heng Wan, Sumitrra Ganguli, Narmadha Meenu Mohankumar, Milan Jain, Kyle Wilson, David Anderson

**Affiliations:** 1 Earth Systems Predictability & Resiliency Group, Pacific Northwest National Laboratory, Richland, WA 99352 USA; 2 Economics, Policy & Institutional Support Group, Pacific Northwest National Laboratory, Richland, WA 99352 USA; 3 Math, Stats & Data Science Group, Pacific Northwest National Laboratory, Richland, WA 99352 USA; 4 Optimization & Control Group, Pacific Northwest National Laboratory, Richland, WA 99352 USA; 5 Risk & Environmental Assessment Group, Pacific Northwest National Laboratory, Richland, WA 99352 USA

**Keywords:** Databases, Society, Databases, Society

Correction to: *Scientific Data* 10.1038/s41597-023-02906-5, published online 18 January 2024

The copyright holder for this article was incorrectly given as ‘The Author(s)’ but should have been ‘Battelle Memorial Institute’.

