# Supplementary material for: Projected income data under different shared socioeconomic pathways for Washington state
Source: Sci Data. 2024 Jan 18;11:85. doi: 10.1038/s41597-023-02906-5 (PMC10796341; doi:10.1038/s41597-023-02906-5)
Supplement: Supplementary file 1 — Supplementary Information [file 41597_2023_2906_MOESM1_ESM.pdf]

# Supplementary Information

## Projected income data under different shared socioeconomic pathways for Washington state

Heng Wan[1], Sumitrra Ganguli[2], Narmadha Meenu Mohankumar[3], Milan Jain[4], Kyle Wilson[2], David Anderson[5]

1. Earth System Predictability & Resiliency Group, Pacific Northwest National Laboratory, Richland, WA 99352, United States
2. Economics, Policy & Institutional Support Group, Pacific Northwest National Laboratory, Richland, WA 99352, United States
3. Math, Stats & Data Science Group, Pacific Northwest National Laboratory, Richland, WA 99352, United States
4. Optimization & Control Group, Pacific Northwest National Laboratory, Richland, WA 99352, United States
5. Risk & Environmental Assessment Group, Pacific Northwest National Laboratory, Richland, WA 99352, United States
corresponding author(s): Heng Wan (heng.wan@pnnl.gov)

Table of Contents:


| | |
|---|---|
| Table S1 | Page 3 |
| Table S2 | Page 4 |

In this study, we have assumed that GDP-to-income ratio is relatively constant over time. To validate this assumption, we obtain historical real GDP (in million dollars) for Washington state from 2011 to 2019. To be consistent with the GDP dollars in the GDP projections dataset we used in this study, we have deflated the real GDP values to 1990 dollars. We download ACS 1-year estimate of total income (in million dollars) for Washington state from 2011 to 2019 and then convert them to 2015 the dollars, to be consistent with the income projections we produced in the future years. Table S1 shows the results of GDP-to-income-ratio between 2011 and 2019. Based on Table S1, we observe that GDP-to-income ratio is relatively constant across years, with a mean ratio of 0.84 and a standard deviation of 0.01.

**Table S1.** Total income, real GDP, and GDP-to-income ratio for Washington state between 2011 and 2019.

| Years | Total income ($M) as in 2015 dollars | Real GDP ($M) as in 1990 dollars | GDP-to-income ratio |
|---|---|---|---|
| 2011 | 207,945 | 246,079 | 0.85 |
| 2012 | 209,012 | 254,193 | 0.82 |
| 2013 | 212,481 | 260,757 | 0.81 |
| 2014 | 219,972 | 269,384 | 0.82 |
| 2015 | 233,711 | 280,480 | 0.83 |
| 2016 | 246,752 | 290,832 | 0.85 |
| 2017 | 259,472 | 305,901 | 0.85 |
| 2018 | 273,189 | 326,858 | 0.84 |
| 2019 | 288,319 | 338,359 | 0.85 |

Another major assumption in this study is that the mean household size remains unchanged over time. To test its validity, we download ACS 1-year estimate of population and number of households for Washington state from 2011 to 2019, and then calculate the mean household size for each year. Table S2 shows the population, number of households, and the calculated mean household size between 2011 and 2019 for Washington state. According to Table S2, Washington has a relatively stable mean household size across years, with a mean of 2.62 and standard deviation of 0.017.

**Table S2.** Population, number of households, and mean household size for Washington state between 2011 and 2019.

| Years | Population | Number of households | Mean household size |
|---|---|---|---|
| 2011 | 6,830,038 | 2,632,621 | 2.59 |
| 2012 | 6,897,012 | 2,636,817 | 2.62 |
| 2013 | 6,971,406 | 2,644,557 | 2.64 |
| 2014 | 7,061,530 | 2,679,601 | 2.64 |
| 2015 | 7,170,351 | 2,728,573 | 2.63 |
| 2016 | 7,288,000 | 2,768,076 | 2.63 |
| 2017 | 7,405,743 | 2,840,377 | 2.61 |
| 2018 | 7,535,591 | 2,895,575 | 2.60 |
| 2019 | 7,614,893 | 2,932,477 | 2.60 |
